# Clonal Hematopoiesis: Impact on Health and Disease

**DOI:** 10.1002/hon.70075

**Published:** 2025-06-15

**Authors:** Francisco Caiado, Markus G. Manz

**Affiliations:** ^1^ Department of Medical Oncology and Hematology University and University Hospital Zurich Comprehensive Cancer Center Zurich Zurich Switzerland

**Keywords:** clonal hematopoiesis, health outcomes, leukemia

## Abstract

The expansion of hematopoietic cell clones, carrying alterations in genes frequently mutated in hematologic malignancies, in the absence of altered hematopoietic cell counts or otherwise defined disease criteria, is termed clonal hematopoiesis (CH). CH is frequently detectable in aged individuals and associates with numerous detrimental health impacts. These impacts are highly dependent on the type of mutations and the cellular context in which they manifest. Mutations in the hematopoietic stem and progenitor cell (HSPC) compartment as well as in self‐renewing more mature cells associate with increased risks of malignant disease, while mutations penetrating via hematopoiesis in non‐self‐renewing, mature cells associate with altered immune functions and consequent systemic effects, which can initiate or aggravate multiple non‐malignant diseases. Here we review the definitions of CH, major genetic drivers and lineage penetrance, and we highlight how CH impacts on hematological and non‐hematological conditions.

## 1 Introduction

The hematopoietic system is responsible for a constant supply of mature hemato‐immune cells that maintain essential organismal functions. To ensure the needed high cellular output, the hematopoietic system is hierarchically organized and sustained by rare hematopoietic stem cells (HSCs), which differentiate in highly proliferative hematopoietic progenitor cells (HPCs), which subsequently give rise to mature hemato‐lymphoid cells. HSCs are localized is specialized bone marrow niches and can maintain their cellular pool size via self‐renewing divisions, while they also give rise to HPCs, which progressively differentiate into mature effector cells. The HSC pool directly contributing to unperturbed hematopoiesis is considered highly polyclonal, with recent estimates of 20,000–200,000 HSCs participating about evenly in leukocyte production in humans below 65 years of age. In contrast, in individuals aged over 75 years, clonal diversity of the hematopoietic system is substantially decreased, with 30%–60% of hematopoiesis accounted for by 12–18 independent HSC clones [1, 2]. This is attributed to both, a loss and suppression of blood production from HSCs due to age‐associated dysfunctions [3] and to the continuous selection and expansion of HSC clones carrying driver‐mutations, rendering them fitter relatively to their non‐mutant counterparts [4]. The result of this life‐long ongoing clonal dynamics is the frequent onset of clonal hematopoiesis (CH, also termed age‐associated clonal hematopoiesis—ARCH), particularly in the aging population. CH associates with increased health risks, predominantly progression into hematological neoplasia (myeloid and lymphoid) but also non‐hematopoietic dysfunctions (cardiovascular, lung, renal, liver, rheumatological diseases), suggesting not only a pre‐leukemic role for CH but also a broader impact in the normal function of the hemato‐immune system [5]. Here we review the fundamental aspects of CH, highlighting its contribution to hematological and non‐hematological neoplasm development, its impact on non‐malignant diseases and implications in current immune‐therapeutic approaches.

## 2 Clonal Hematopoiesis (CH): Definition, Incidence and Drivers

**FIGURE 1:**
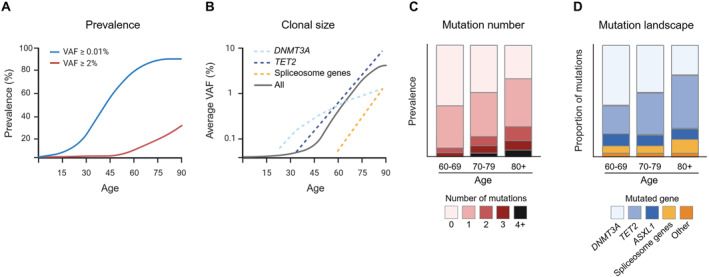
Age associates with key properties of clonal hematopoiesis (CH). (A) The estimated prevalence of CH as a function of age varies according to the variant allele frequency (VAF) used as a threshold for CH definition, which in turn depends on the sequencing method used. (B) Representative depiction of the average clonal size as a function of age (solid gray line), indicating increased clonal sizes in advanced ages. Mutation‐specific clonal expansions are indicated (dashed lines), with *DNMT3A‐*mutant clones showing higher expansion rates in earlier stages, *TET2‐*mutant clones showing a later onset and then a constant life‐long expansion rate, and spliceosome gene‐mutated clones showing a late onset and the highest expansion rates [7]. The category of spliceosome mutations includes SF3B1, SRSF2 and U2AF1. (C) Representative depiction of the prevalence of mutational number per individual according to age category, indicating increased mutational number in advanced ages (graph adapted from published data [8]). (D) Representative depiction of the proportion of mutations in different driver genes among all mutations detected per age category, indicating reduced relative frequency of *DNMT3A* mutations and increases in *TET2* and *ASLX1* mutations in advanced age. The category of spliceosome mutations includes SF3B1, SRSF2 and U2AF1 (graph adapted from published data [8]).

According to the World Health Organization (WHO) classification, clonal hematopoiesis (CH) refers to the presence of a population of cells derived from a mutated hematopoietic multipotent stem/progenitor cell harboring a selective growth advantage in the absence of unexplained cytopenias, hematological cancers, or other clonal disorders [6]. CH encompasses a complex group of entities that are defined based on the knowledge of the identity of the driver gene, the variant allele fraction (VAF) of the driver gene, the type of gene mutated and the presence or not of mosaic chromosomal alterations (mCAs) [5]. We here focus on the most frequent form of CH: clonal hematopoiesis of indeterminate potential (CHIP), defined by the presence of one or more somatic mutations in known myeloid leukemia driver genes, detected in the blood or bone marrow cells at a VAF equal or > 2% (or over 4% for X‐linked gene mutation in males) in individuals lacking diagnostic criteria for defined hematological disorder or unexplained cytopenia [6]. Recent estimates suggest that incidence of CHIP in the general adult population (40–70‐year‐old) is around 5% and increases to 10%–20% in individuals over 70 years (Figure 1A). The majority (∼90%) of CHIP carriers have a single mutation, with mutations in one of three genes involved in epigenetic regulation: *DNMT3A, TET2*, and *ASXL1* (DTA), accounting for two‐thirds of recurrent mutations, with the remaining one third including mutations in additional known myeloid neoplasia driver genes such as *JAK2, TP53, GNAS, PPM1D, BCORL1*, and *SF3B1* [9, 10, 11]. Importantly, not only incidence increases with age, but also mutant clonal size and complexity (defined by the presence of more than one mutation) (Figure 1B,C). Interestingly, and contrasting with the definition of CH, CHIP cases that display one or more persistent unexplained cytopenias (and therefore a non‐normal health state) are classified by the WHO as clonal cytopenia of undetermined significance (CCUS) [6]. Compared with CHIP, CCUS has an overlapping mutational landscape, an approximate 10‐fold lower incidence, higher clonal size and clonal complexity and an increased risk of progression to myeloid neoplasia (MN—encompassing acute myeloid leukemia, myelodysplastic syndromes and myeloproliferative neoplasms) compared to CHIP [12, 13, 14], suggesting that CCUS likely represents a developmental intermediate state between CHIP and MN. We here use CH as an umbrella term for both CHIP and CCUS, as those two states are not always clearly separated in prior studies we relate to in this review.

## 3 Lineage‐Specific Mutational Landscape of CHIP

CHIP was initially predominantly perceived as a myeloid form of clonal hematopoiesis. This is attributed to the fact that per definition the mutations first looked for and identified in CHIP carriers are frequently found in myeloid malignancies, and to the subsequent confirming observation that most CHIP carriers have an increased risk of developing myeloid but not lymphoid neoplasms [15]. In agreement, when looking for the penetrance of mutations in mature hematopoietic populations in CHIP carriers, it has been shown that mutant VAFs are higher in monocytes, granulocytes, and NK‐cells compared to B‐ or T‐cells. Moreover, when looking into bone marrow samples, CHIP mutations can be found in Lin^−^CD34^+^CD38^−^ HSPCs with subsequent expansion to myeloid primed progenitors, strongly suggesting that CHIP derives from mutated HSPCs and then propagates preferentially into the myeloid branch, with lower penetration into and/or expansion within the lymphoid compartment. This might be due to a reduced permissiveness of some CHIP mutations for the complex process of B‐ and T‐cell development, as well as the overall reduced B‐ and T‐cell development in adult life, where some of the respective homeostasis is driven by mature B‐ and T‐cells [16, 17]. Interestingly, CH has also been recently proposed to precede the onset of lymphoid malignancies [15]. This entity is substantially less frequent than myeloid CHIP and will be discussed briefly at the end of this review.

## 4 CH and Progression to Hematological Neoplasm

CH is perceived as a pre‐malignant state of the hematopoietic system, as the somatic mutations detected in CH are frequently the founding mutations in hematologic malignancies. Indeed, CHIP and CCUS are both associated with an approximate 10–12 fold increased risk of MN development (the risk is slightly higher for CCUS) [18], with an overall rate of transformation of 0.5%–1% per year. However, this risk is highly dependent on specific characteristics. These include: (1) mutated gene—single mutation in *DNMT3A* has the lowest risk, while mutations in splicing factor genes (*SF3B1, SRSF2*), *TP53, IDH1, IDH2*, and *RUNX1* have the highest risk; (2) number of mutations within a clone—higher mutational number (> 2 mutations) poses a higher risk; (3) mutant clone size—higher clonal sizes (VAFs ≥ 20%) pose a higher risk; (4) red cell characteristic—red cell distribution width (RDW) of 15% or more and mean corpuscular volume (MCV) of 100 fL or more associate with increased risk; (5) The onset of one or more persistent cytopenias—individuals with CCUS have higher risk than individuals with CHIP; and (6) age—individuals over 65 have increased risk [15]. Interestingly a recent study further highlights that mutational landscapes in CHIP carriers associate with increased propensity to develop specific myeloid malignancies, in detail, *SF3B1* and *SRSF2/TET2* co‐mutated cases associate with higher risk of MDS, and SRSF2/IDH2 co‐mutated cases are more likely to develop AML. Also, *DNMT3A*‐R882 mutations are specifically associated with AML [19]. Overall, ongoing efforts combining different CH parameters to establish myeloid neoplasia transformation risk scores are essential to understand not only the biology of the transition between pre‐ and full‐blown malignancy, but also hold the potential for a more precise staging and clinical management of CH carriers.

## 5 CHIP and Non‐Malignant Disease

**FIGURE 2:**
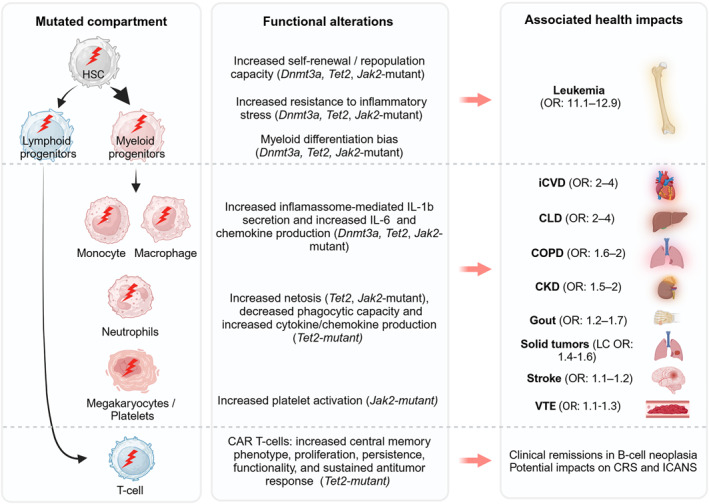
Health impacts of CHIP. Schematic depiction of the cellular compartment affected by CHIP‐associated mutations (left), the reported functional cellular alterations that associate with cell‐type specific CHIP mutations (middle) and the reported health impacts associated with CHIP (right). Red arrows between middle and right box indicate association but not causality. CAR, chimeric antigen receptor; CKD, chronic kidney disease; CLD, chronic liver disease; COPD, chronic obstructive pulmonary disease; CRS, cytokine release syndrome; HSC, hematopoietic stem and progenitor cell; ICANS, immune effector cell‐associated neurotoxicity syndrome; iCDV, ischemic cardiovascular disease; LC, lung cancer; OR, odds ratio; VTE, venous thromboembolism.

A growing body of evidence indicates that CHIP mutation‐specific effects in the mature hematopoietic compartment can lead to immune cell dysfunction and associated increased risk of nonmalignant disorders and overall mortality [20]. Indeed, multiple age‐related inflammatory pathologies have been positively associated with CHIP and likely act as a disease‐modifying factor. These include cardiovascular disease [21], chronic liver disease [22], chronic kidney disease (CKD) [23], chronic obstructive pulmonary disease (COPD) [24], gout [25], osteoporosis [26], stroke [27], and venous thromboembolism (VTE) [28] (Figure 2). Interestingly, CHIP has only been shown to be protective of one disease, Alzheimer's dementia, however the underlying mechanism thus far remains elusive [29].

After leukemic transformation, ischemic cardiovascular disease (iCVD) is the pathology with higher risk in CHIP carriers, with an estimated 2‐fold increased risk of coronary heart disease and a 4‐fold increased risk of myocardial infarction in CHIP carriers compared to non‐carriers, with the strongest associations observed for *TET2‐* and *JAK2‐*mutant cases. Importantly, and as observed for the risk of myeloid malignancy progression, clone size is also prognostic of iCVD outcomes in CHIP, with individuals with a VAF ≥ 10% showing higher iCVD risk than those with smaller clones [21]. Mechanistically, studies in mouse models attribute this effect to increased pro‐inflammatory cytokine production (IL‐1β, IL‐6, and chemokines) by *Dnmt3a/Tet2‐*mutant myeloid cells, particularly in the context of atherosclerotic plaque formation [30, 31]. Consistent with this finding, exploratory analysis of the IL‐1β inhibitor “canankinumab anti‐inflammatory thrombosis outcomes study” (CANTOS) showed reduced cardiovascular events, particularly among individuals with *TET2‐*mutant CHIP [32]. Liver disease is also highly impacted by CHIP, with VAF ≥ 10% doubling the risk of chronic liver disease (CLD), nonalcoholic hepatic steatosis, and cirrhosis in CHIP carriers. Also, this association is highly dependent on the type of CHIP mutations, with increased risk for CLD of 17.6‐fold for *JAK2*‐mutant CHIP, 5.4‐fold increase for *TET2*‐mutant CH, and a low risk for *DNMT3A* mutations. Mouse models of *Tet2*‐mutant CHIP studies further implicate liver resident macrophage‐derived inflammasome‐dependent inflammation (IL‐6, CXCL1, CCL22, and CCL17) in this outcome [22]. The impact of CHIP on the incidence of (chronic kidney disease (CKD), chronic obstructive pulmonary disease (COPD), gout, osteoporosis, stroke and venous thromboembolism (VTE) are more moderate. For CKD, CHIP leads to 2.2‐fold risk of kidney failure over 5 years of follow‐up and increased risk of complications related to CKD, including kidney disease in context of anemia [33]. For COPD, individuals with CHIP show risks of moderate‐to‐severe, severe, or very severe COPD that were 1.6 and 2.2 times greater than those of noncarriers. Mouse *Tet2*‐mutant CHIP models with COPD further show that *Tet2* loss in hematopoietic cells enhances pulmonary inflammation, increases IFN signaling, decreases TGF‐β signaling, and accelerates the development of emphysema [24]. In the case of gout, CHIP with a VAF ≥ 10% was associated with 1.3 times the risk of incident compared with no CHIP after adjusting for common gout risk factors. This risk was particularly higher in *TET2*‐mutant CHIP. In vivo mechanistic studies further revealed that *Tet2*‐knockout mice have increased monosodium urate‐induced inflammation in an NLRP inflammasome–dependent manner due to the greater production of IL1β by macrophages. As for osteoporosis, the risk of incident in individuals with CHIP is 1.4 times that of non‐CHIP. Moreover, larger CHIP clones, especially in *DNMT3A*, with VAF ≥ 10% were significantly correlated with lower estimated bone mineral density, consistent with accelerated bone loss. Mechanistically, this was associated with proinflammatory cytokines, including Irf3‐NF‐κB–mediated IL‐20 expression from *Dnmt3a*‐mutant macrophages [26]. Overall, the CHIP‐associated and age‐associated inflammatory diseases align with findings in mice, in which particularly *Tet2*‐ and *Dnmt3a*‐mutated compartments contribute to enhanced inflammatory settings [34, 35, 36, 37, 38].

## 6 CHIP and Solid Tumors

The associations between CHIP and solid tumors were initially revealed in cohorts of patients under treatment for solid malignancies. Accordingly, an initial study in a cohort of 8810 patients treated at Memorial Sloan Kettering Cancer Center, identified CHIP in 25% of analyzed patients. Moreover, CHIP carriers with higher clonal sizes (VAF > 10%) had a reduced overall survival with the most common cause of death being progression of the primary tumor [39]. These associations have been generally confirmed in subsequent studies [40]. However, such analysis on patients undergoing therapy poses a high confounding risk, as (chemo)therapies can be direct drivers of CHIP (particularly in mutations in DNA damage response pathway genes [41]) or give further advantage to the mutated clones, and due to the existence of shared CHIP and cancer risk factors (e.g., age, smoking), which limit the ability of these studies to make casual conclusions about the risk associations between CHIP and cancer. These limitations have been reduced in larger, non‐cancer specific longitudinal studies. An analysis of 200,453 individuals from the UK Biobank found that CHIP, especially with mutation VAF > 10%, associates with lung cancer, kidney cancer, lymphoma, and sarcoma incidence. Interestingly, *DNMT3A*‐mutant CHIP also associated with incidence of stomach and bladder cancer, while mutations in splicing factors *SF3B1* and *SRSF2* were additionally associated with higher rates of colorectal and head/neck cancers [42]. A similar analysis using 628,388 individuals found relationships between CHIP and incident risk of lung, skin, prostate and breast cancer [43]. Indeed, lung cancer seems to be the solid tumor type mostly influenced by the presence of CHIP. CHIP (particularly in *DNMT3A*, *TET2*, and *ASXL1* genes, with VAFs > 10%) associates with a 36% risk increase for lung cancer across several cohorts, even when controlling for other confounders [44]. Mechanistically, it is likely that mutant cells contribute directly to a dysfunctional tumor microenvironment (TME), as different studies have identified mutant immune cells in the TME in human solid tumor samples [45, 46]. Concerning the effects of hematopoietic *Tet2* loss of function in tumor progression the reports are mixed. One such study, using a full *Tet2* KO mouse model, suggests a pro‐tumorigenic effect of *Tet2* deletion via an IL‐6‐mediated immunosuppressive effect, which promotes tumor growth in models of hepatocellular carcinoma and breast cancer [47]. Also, models of hematopoietic *Tet2* deletion suggests that *Tet2*‐mutant myeloid cells produce higher levels of S100a8/S100a9, which enhance vascular endothelial growth factor A (VEGF‐A) production by cancer cells, leading to increased tumor vasculature and growth in subcutaneously transplanted lung cancer [48]. Interestingly, in melanoma *Tet2*‐mutant CHIP effects seem to act contrary, resulting in reduced tumor burden via a *Tet2*‐loss mediated onset of proinflammatory tumor‐associated macrophage phenotype that promotes T‐cell infiltration [49]. Clearly, the full scope of how different CHIP drivers impact on tumor biology in different contexts needs more granular resolution (*Dnmt3a* [50], *Asxl1* [51], and p53 [52]).

## 7 CHIP and Immunotherapy/Blood Cancer Therapy

Hematopoietic stem cell transplantation (HSCT) is currently either applied in an autologous (autologous‐HSCT) or in an allogeneic setting from sufficiently HLA‐compatible donors (allo(geneic)‐HSCT). Autologous‐HSCT is generally used to bridge hematopoietic failure after cancer treatment with aggressive chemotherapy, while allo‐HSCT is used to treat congenital or acquired marrow failure, and, in most cases, to exploit the graft versus tumor effect of donor allogeneic cells against recipient hematologic malignancies. CHIP is frequently observed in patients undergoing HSCT (reviewed in [53]). CHIP incidence in autologous‐HSCT is reported in the range of 10%–30% post transplantation (with older patients carrying higher CHIP incidence) and associates with enrichment of mutations in DNA‐damage response genes (mostly in *TP53* and *PPM1D*) [54, 55, 56, 57]. This is likely a consequence of autologous‐HSCT patients being exposed to chemotherapy previously to graft collection, which selects for pre‐existing mutations conferring DNA‐damage resistance, and a consequence of subsequent proliferative advantage in a reduced hematopoietic compartment. Notably, the presence of pretransplant CHIP in autologous‐HSCT patients associates with increased risk for development of so called “therapy‐related” myeloid neoplasms (t‐MNs) and inferior overall survival, driven by t‐MNs and by non‐relapse‐associated adverse events [58]. CHIP incidence in allo‐HSCT donors ranges from 16% to 24% and the mutational spectrum overlaps with CHIP in the general population. Donor CHIP mutations are frequently found in recipients, where they show consistent but modest expansions [59, 60, 61]. Indeed, two models of HCT‐specific selection have been proposed, with pruning selection referring to cases when cell divisions occur mostly in the donor pre‐transplantation and consequent clonal dominances are maintained in the recipient. While in growth selection, which is more frequent in clones with multiple driver mutations, cell divisions and consequent clonal dominances occur in the recipient after engraftment [62]. Importantly, while donor derived leukemia can occur at low rates, typically from donor CHIP with *TP53* or splicing factor mutations or from donors carrying germline *DDX41* mutations [61], donor‐engrafted CHIP after allo‐HSCT associates with decreased risk of disease relapse but does not affect overall survival, progression‐free survival or non‐relapse mortality [58]. Strikingly, specifically donor *DNMT3A*‐CHIP (at VAF > 1%) associates with improved recipient survival due to reduced relapse risk and concomitant increased risk for chronic graft‐vs‐host disease which associates with an increased inflammatory milieu (mostly IL‐12) in recipients [61]. A similar phenomenon is observed in the context of CAR T‐cell therapies, where the disruption of the CHIP‐associated genes *TET2*, *DNMT3A*, and *CBL in* CAR T‐cells have been associated with clinical remissions. In these cases, CAR T‐cells carrying these alterations showed higher central memory phenotype, resulting in higher T‐cell proliferation, long‐term persistence, increased functionality, and sustained antitumor response [63, 64, 65]. On the other hand, the impact of CAR T‐cell therapies on cancer patients that carry CHIP seems to be more heterogenous and probably disease dependent, with studies suggesting no impact [66], increased rates of complete response and cytokine release syndrome (CRS) severity in patients younger than age 60 years [67], or increased risk of immune effector cell‐associated neurotoxicity syndrome (ICANS), CRS severity, and higher cumulative incidence of therapy‐related myeloid neoplasms after CAR T‐cell therapy [68]. Overall, these studies indicate that further investigation into the mechanisms and interventions to guide CAR T‐cell therapy in the context of CH are warranted.

## 8 Lymphoid Clonal Hematopoiesis and Lymphoid Neoplasia

**FIGURE 3:**
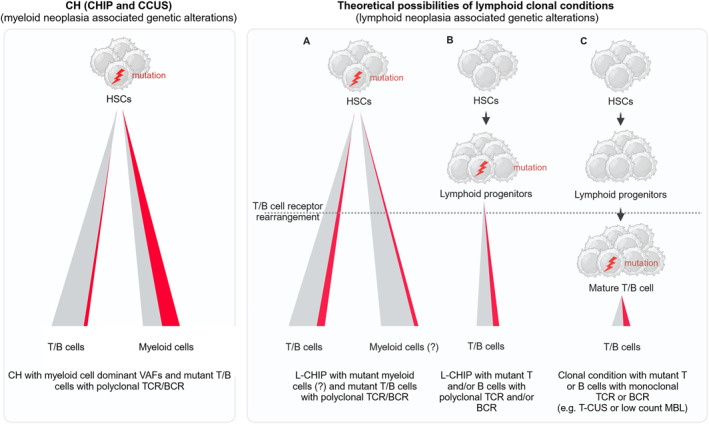
Cell of origin in clonal conditions of the hematopoietic system. Schematic depiction of the implications of the cell of origin in hematopoietic clonal conditions. In CH (CHIP and CCUS) (left panel), the mutations are thought to occur at the HSC level leading to higher variant allele frequency detection in the mature myeloid compartment, with minor presence of the mutation in the lymphoid compartment. In lymphoid clonal conditions (right panel), the mutations can occur in one of three scenarios: (A) Mutation occurrence in the HSC compartment with transmission to lymphoid (and presumably to myeloid) progeny, which will then undergo receptor rearrangement to form a T/B cell receptor‐diverse mutant clone. How L‐CHIP mutations propagate in the myeloid lineage is currently largely unknown (depicted with question mark). (B) Mutation occurrence in the lymphoid progenitor compartment before receptor rearrangement, leading to a T/B cell receptor‐diverse mutant clone. (C) Mutation occurrence at lymphocyte developmental stages, which have already undergone T‐ or B‐cell receptor rearrangement, which upon expansion form a monoclonal T‐ or B‐cell receptor mutant clone. The distribution of mutations in other lineages (e.g., natural killer cells) has been omitted for simplicity. MBL, monoclonal B lymphocytosis; T‐CUS, T‐cell clones of uncertain significance; VAF, variant allele frequency.

CH was originally defined by sensitive genetic testing for the presence of known myeloid neoplasia driver genes in hematopoietic healthy appearing populations [9, 10, 11]. Taking a similar approach, but focusing on sensitive testing for the presence of known recurrent lymphoid neoplasia driving genes as well as on mosaic chromosomal alterations (mCA), Niroula et al. recently subdivided CH in myeloid CHIP (M‐CHIP) and lymphoid CHIP (L‐CHIP), as well as myeloid mosaic chromosomal alterations (M‐mCAs) and lymphoid mosaic chromosomal alterations (L‐mCA) [15]. Both L‐CHIP and L‐mCA are defined by a 2% VAF in blood leukocytes without meeting diagnostic criteria of a lymphoid malignancy. Like CHIP and CCUS, the prevalence of L‐CHIP increases with age, but is less common than its myeloid counterpart with a predicted incidence of approximately 1% in the adult population (40–70 years old), that is in the elderly population about a log less prevalent than M‐CHIP. The mutational landscape of L‐CHIP includes genes most frequently implicated in lymphoid malignancy, and contrarily to M‐CHIP, there is an even distribution of genotypes without a clear set of dominant mutations. Interestingly, while L‐CHIP associates with increased incidence of lymphoid malignancy (mostly chronic lymphocytic leukemia and small lymphocytic lymphoma) there is no association with coronary artery disease or overall mortality, as observed for M‐CHIP [15]. Concerning the clonal population driving L‐CHIP, it is expected that the mutation occurs in HSCs and lymphoid progenitors, however formal proof of this is still lacking. Importantly L‐CHIP should be distinguished from other mature lymphoid cell clonal conditions. Indeed, the long‐term persistence of mature T‐ and B‐cells (with uniquely rearranged T‐ or B‐cell receptors), coupled with their proliferative capacity and programmed mutagenesis upon antigen recognition (in B‐cells) creates additional opportunities for these populations to acquire L‐CHIP mutations. Thus, caution in classification of these cases as L‐CHIP is recommended, as they might represent conditions such as (very) low count monoclonal B lymphocytosis (MBL) or T‐cell clones of uncertain significance (T‐CUS) [69] (Figure 3). Moreover, mutations occurring at the stem and progenitor level and their fixation in the lymphoid system might depend on the fitness gains they provide to specific cellular states along the hematopoietic tree, and possibly secondary mutations occurring at more mature, self‐renewing cell states. Concerning L‐CHIP, the full scope of clonal metrics predisposing for transformation are still undetermined. However clonal size and higher mutational numbers associate with increased risk of malignancy, particularly in individuals with elevated lymphocyte counts [15]. L‐CHIP connects to subsequent educational presentations on clonal T‐cell, B‐cell and plasma cell precursor states.

## Conflicts of Interest

The authors declare no conflicts of interest.

## Peer Review

The peer review history for this article is available at https://www.webofscience.com/api/gateway/wos/peer-review/10.1002/hon.70075.

## Data Availability

The authors have nothing to report.
